# Time to death and its predictors among neonates admitted with sepsis in neonatal intensive care unit at comprehensive specialized hospitals in Northeast Ethiopia

**DOI:** 10.3389/fped.2024.1366363

**Published:** 2024-04-17

**Authors:** Muluken Amare Wudu, Yemane Eshetu Bekalu, Endalk Birrie Wondifraw, Tarikua Afework Birhanu, Molla Kassa Hailu, Melaku Ashagire Belete, Yaregal Semanew Endeshaw

**Affiliations:** ^1^ Department of Pediatrics and Child Health Nursing, College of Medicine and Health Sciences, Wollo University, Dessie, Ethiopia; ^2^ Department of Public Health, ALKAN Health Sciences and Business College, Dessie, Ethiopia; ^3^ Department of Medical Laboratory Science, College of Medicine and Health Sciences, Wollo University, Dessie, Ethiopia

**Keywords:** time to deaths, predictors, neonatal sepsis, comprehensive hospitals, Northeastern Ethiopia

## Abstract

**Background:**

In impoverished nations like Ethiopia, neonatal sepsis contributes significantly to neonatal mortality. Despite variations in the specific timing of death and predictors of neonatal mortality associated with sepsis across different settings, there's limited documented information in the Neonatal Intensive Care Units of northeastern Ethiopia. Consequently, the aim of this study was to determine time to death and its predictors among neonates with sepsis admitted to Neonatal Intensive Care Units in comprehensive specialized hospitals in northeastern Ethiopia

**Methods:**

A prospective cohort study conducted at the institution level involved 306 neonates diagnosed with sepsis. Data collection utilized face-to-face interviews and chart reviews. Subsequently, the data were inputted into Epi-data version 4.6 and later analyzed using STATA version 17. The median time to death was determined, and both the Kaplan-Meier curve and log-rank test were applied. Furthermore, a Cox proportional hazard regression model was utilized to identify predictors of neonatal mortality associated with sepsis.

**Result:**

The cumulative incidence of mortality among neonates admitted with sepsis was 34% (95% CI: 28.9%–39.5%). The neonatal mortality rate stood at 51 (95% CI: 42.1, 62) per 1,000 neonates admitted to the intensive care units with sepsis over a total of 1,854 person-days of observation. Additionally, the median time to death was 13 days (IQR = 5–23 days). Tachypnea [AHR 6.2 (95% CI: 1.5–9.7)], respiratory distress syndrome [AHR 2.1 (95% CI: 1.3–3.5)], lethargy [AHR 1.8 (95% CI: 1.2–2.6)], preterm birth [AHR 1.8 (95% CI: 1.2–2.7)], continuous positive airway pressure use [AHR 2.1 (95% CI: 1.3–3.4)], home delivery [AHR 2.63 (95% CI: 1.1–6.4)], Subgalea hemorrhage [AHR 1.8 (95% CI: 1.1–3.9)], and low platelet count [AHR 5.9 (95% CI: 2.3–8.6)] were found to be predictors of time to death in neonates with sepsis.

**Conclusion:**

The study revealed an alarmingly high neonatal mortality rate among septic neonates, underscoring the urgency for intervention. Enhancing the quality of care in neonatal intensive care units, bolstering infection prevention during procedures such as continuous positive airway pressure, exercising caution with locally made accessories, and reinforcing a culture of institutional delivery are critical in curbing neonatal sepsis-related mortalities.

## Introduction

Neonatal sepsis is an infection that emerges within the first 28 days of a newborn's life. It is characterized by a systemic inflammatory response syndrome triggered by a suspected or confirmed infection (1, 2). Neonatal sepsis can manifest in two distinct time frames: early-onset sepsis (EOS) occurs within the first 7 days of life, typically within 72 h, and may be acquired during prenatal or intrapartum periods. Conversely, late-onset sepsis (LOS) emerges between 8 and 28 days after birth and is primarily acquired through environmental exposure (1). Additionally, neonatal sepsis can manifest with various presentations, ranging from subclinical infection to severe localized or systemic signs such as convulsions, respiratory distress, poor feeding, lethargy, and jaundice (3).

The health of newborns is seriously affected by neonatal sepsis, and consequently, neonatal sepsis caused 0.23 million deaths worldwide and 6.31 million incidence cases of the condition in 2019. From 1990 to 2019, there were global trends of rising incidence and falling mortality from newborn sepsis, with sub-Saharan Africa and Asia bearing the greatest absolute burden (4). Western Sub-Saharan Africa posed the greatest threat to neonatal sepsis mortality (8.18 per 100,000), while Eastern Sub-Saharan Africa came in second (6.88 per 100,000), including Ethiopia in 2019 (5).

Neonatal sepsis in Ethiopia ranks among the top three causes of neonatal mortality, contributing to one-third of all neonatal deaths (6). Studies conducted in Ethiopia have reported varying incidence rates of neonatal sepsis death, ranging from 8.65 per 100 neonates to 28 per 1,000 neonates in southern and northwest regions, respectively (7, 8). Consequently, Ethiopia, ranking fourth globally in neonatal mortality (6) and among over 60 nations, including Ethiopia, faces challenges in achieving the 2030 Sustainable Development Goals (SDGs) for reducing neonatal mortality (9).

Neonatal sepsis is estimated to have resulted in the loss of between 5.3 and 8.7 million disability-adjusted life years, along with complications such as disseminated intravascular coagulation (DIC), respiratory failure, acute renal failure, multiorgan dysfunction, and neurodevelopmental issues. Moreover, it has led to approximately half a trillion dollars in lost economic output in sub-Saharan Africa alone (10–12).

Prior research has established an association between neonatal sepsis and various clinical characteristics of the newborn, such as preterm birth, low birth weight, respiratory distress syndrome (RDS), duration of labor, and perinatal hypoxia. Additionally, neonatal sepsis has been associated with factors including maternal age, income level, antenatal care, maternal fever before labor, sexually transmitted infections, initiation of breastfeeding, birth resuscitation, and nasogastric tube (NGT) insertion (3, 7, 8, 13–16).

Ethiopia has pledged to achieve the SDGs and has implemented substantial measures to reduce neonatal mortality. These include the Integrated Management of Newborn and Childhood Illness (IMNCI), Kangaroo mother care (KMC), improvement of neonatal intensive care unit (NICU) services in hospitals, capacity building for care providers, neonatal resuscitation training, and the use of empirical antibiotics (17).

While previous cohort studies have explored time to death and its predictors among neonates with sepsis in Ethiopia (7, 8), they primarily relied on retrospective and single institution-based methodologies, indicating a methodological gap. In contrast, our study adopted a prospective cohort design, encompassing multiple centers and incorporating additional variables like birth injuries and respiratory support types. This study aims to determine time to death and predictors among neonates with sepsis admitted to NICUs in comprehensive specialized hospitals in northeastern Ethiopia, aiming to bridge information gaps and offer recommendations to local health stakeholders. Ultimately, our findings can empower hospital managers, care providers, and institutions to enhance neonatal sepsis care and foster survival rates. Furthermore, our study may inspire further multi-level analysis research by other scholars.

## Methods

### Study period, design, and setting

A multicenter prospective follow-up study was conducted from August 8 to 27 October 2023, in the Neonatal Intensive Care Units (NICUs) of three comprehensive specialized hospitals in northeastern Ethiopia: Woldiya Comprehensive Specialized Hospital, Dessie Comprehensive Specialized Hospital, and Debrebrehan Comprehensive Specialized Hospital. These hospitals collectively serve more than ten million people. Each hospital maintains an average of 33 beds, with 203 patients admitted monthly. The nurse-patient ratio stands at 1:4, while the physician-patient ratio is 1:9 in the NICU. All hospitals provide continuous positive airway pressure (CPAP), mechanical ventilation, and phototherapy services.

### Population and eligibility criteria

All neonates diagnosed with neonatal sepsis and admitted to the NICU in comprehensive specialized hospitals in northeastern Ethiopia constituted the source of population. However, neonates with sepsis randomly selected from those admitted to the NICU in these hospitals were included as study units.

This study comprised all neonates diagnosed with neonatal sepsis who were hospitalized in the NICU and had complete sociodemographic responses, along with comprehensive documentation of newborn clinical features, diagnostic studies, management provided, and hospitalization outcomes. However, neonates with major congenital anomalies requiring immediate surgical and medical interventions were excluded.

### Sampling size determination and sampling procedures

The sample size was determined by using the double population exposure difference formula using STATA version 17 by taking into account the following assumptions: each covariate hazard ratio (HR) and the probability of event (PE) for the factors, a variability (SD) of 0.5, a confidence interval (CI) of 95%, an 80% power with ratio (1:1), and the probability of withdrawal of 10%. Based on this, the low-birth-weight HR and the PE were (2.12, 28.1%) with a 221-sample size, preterm birth (HR = 2.06, PE = 33.5%) with a 200-sample size, respiratory distress syndrome (HR = 1.77, PE = 35.5%) with a 306-sample size, and oxygen saturation less than 90% (HR = 2.23, PE = 26.4%) with a 209-sample size, as taken from a previous study (8). As a result, the maximum sample size was 306.

In the previous three months, the average number of neonates admitted with sepsis was 971 (Dessie Hospital = 348, Woldiya Comprehensive Hospital = 303, and Debrebrehan Comprehensive Hospital = 320). The required number of subjects was then allocated proportionally to each selected hospital based on population size. Accordingly, 110 newborns from Dessie Hospital, 96 from Woldiya Comprehensive Hospital, and 100 from Debrebrehan Comprehensive Hospital were included in the study. The neonatal logbook, containing the neonatal medical register number, served as the sample frame. Finally, neonates with sepsis were selected from each hospital using systematic random sampling approaches at every third interval until the desired sample size was achieved, with the first neonate chosen by a lottery system (where the neonate's medical registration number was considered a code).

## Variables of the study

### Dependent variable

Time to death of neonates with sepsis.

### Independent variables

Socio-demographic factors (e.g., age of mothers, sex and age of neonates, educational level of mothers, and occupational status).

Obstetrics, labor, and delivery factors (e.g., parity, history of ANC, history of intrapartum fever, prolonged labor, mode and place of delivery, premature rupture of membranes, and pregnancy-related or chronic medical problems).

Newborn clinical and diagnostic features (e.g., gestational age, weight of the neonate, respiratory status, temperature status, RDS, PNA, type of sepsis, jaundice, breastfeeding, reduced movement, lethargy, WBC count, platelet count, and gram stain result).

Complications and management of sepsis (e.g., severe anemia, thrombocytopenia, septic shock, type of respiratory support, antibiotics, maintenance fluid, antiseizure, counseling on newborn care).

### Operational definition

**Neonatal sepsis** is diagnosed as if the neonates have at least more than one of the following clinical features: inability to breastfeed, convulsions, movement only when stimulated, unconsciousness, fast breathing rate (60 breaths per minute), body temperature greater than 37.5°C or <35.5°C, tachycardia (>160 bit/min) or bradycardia (<100 bit/min), and desaturation (<90% oxygen saturation). Furthermore, at least two hematological criteria for abnormality exist, such as a total leukocyte count <5,000 or >12,000 cells/µl and a platelet count <150 × 103 or >450 × 103 cells/μl (18).

**Early-onset neonatal sepsis** is defined as sepsis identified in neonates within the first seven days of birth (18).

**Late-onset neonatal sepsis** is defined as sepsis diagnosed in newborns aged more than seven days to 28 days after birth (18).

**Neonatal meningitis** is an inflammation of the meninges that occurs within the first 28 days of life and is diagnosed when neonates exhibit at least one of the following clinical features: abnormal neurological examination including seizures, abnormal tone, and full fontanels (18).

**Death**: Neonate with sepsis who passed away during hospitalization within 28 days of delivery and had causes of death reports.

**Censored**: Neonates with sepsis who were referred to another facility, left without medical recommendation, and discharged due to improvement.

**Follow-up time:** From the time of enrollment with a neonatal sepsis diagnosis until individuals died or were censored within 28 days after birth.

**Time to death**: The number of days it takes from NICU admission until the neonate with sepsis dies during the follow-up within 28 days after birth.

**Respiratory distress syndrome (RDS)**: is diagnosed as if the neonates have at least more than two of the following clinical features: grunting, flaring, retraction, and tachypnea, cyanosis, and oxygen saturation less than 90% (18).

**CPAP (Continuous Positive Airway Pressure)** local accessories are utilized in study areas with limited resources. These accessories serve to replace the CPAP tube package and are crafted from materials such as plastic from normal saline bags or, more commonly, repurposed water bottle containers. These containers are filled with water, forming bubbles whose depth is accurately measured and labeled in centimeters. Primarily designed for the care of preterm infants, these accessories play a crucial role in maintaining lung expansion and preventing alveolar collapse during expiration. The pressure required for CPAP is sustained by the water bubbles within the plastic container, ensuring effective respiratory support for these vulnerable patients. Despite the Ethiopian national guidelines not mentioning locally made CPAP accessories, healthcare providers have taken proactive measures to address the widespread resource constraints in the country (Supplementary File).

### Data collection methods and procedures

The data gathering tool was developed based on national technical guidance for maternal and prenatal death surveillance and response (19, 20), the national neonatal intensive care unit guideline follow-up sheet (18), and various literature sources (7, 8, 15). Data collection involved an interviewer-administered questionnaire, direct face-to-face interviews with mothers, and document reviews. Socio-demographic and certain obstetric variables were obtained through direct face-to-face interviews with newborn mothers. Additionally, an observational checklist was used to collect obstetrics and delivery-related variables, essential neonatal characteristics, clinical and diagnostic features, sepsis management and complications, and sepsis outcomes. Information was extracted from the neonatal chart and through daily follow-up.

The validity was subsequently confirmed through expert discussions involving pediatricians, neonatal nurses, pediatric nurses, and public health specialists (Epidemiology and Biostatistics), along with a pre-test study. Revisions were then made based on the pre-test and input from experts to ensure that the questionnaire accurately assessed its intended objectives. The content validity ratio (CVR) and content validity index (CVI) were then calculated, yielding values of 0.80 and 0.84, respectively, indicating the instrument's validity. Following data collection via a pre-test study, the questionnaire underwent evaluation for reliability (reliability coefficient = 0.789, indicating satisfactory reliability) and was assessed for subject appropriateness, simplicity, order, and flow. Initially developed in English, the data collection questionnaire was subsequently translated into Amharic and then back into English to ensure accuracy and uniform understanding.

Data were collected in each hospital's NICU by two neonatal nurse data collectors, each supervised directly by a physician in addition to the investigator. The data collectors monitored the neonates' status upon admission until the end of follow-up or the occurrence of the event of interest (all assessments were completed, and data were obtained). Neonates were examined, and relevant data were collected during each follow-up visit, including neonatal measurements, clinical characteristics, and diagnostic and laboratory test findings. Additionally, any essential management, drugs, or procedures administered during the medication period were documented, and the neonates' outcome status was assessed.

### Data quality assurance and management

The data collectors and supervisors underwent initial training and received ongoing monitoring. Written consent was obtained from the mothers of each newborn, explaining the study's objectives and significance. Furthermore, immediate supervisors reviewed the obtained data for accuracy and completeness daily. Crucially, the study authors pretested the questionnaire in a similar environment, covering 5% of the total sample size at Kombolicha General Hospital, which was not part of the primary study. Following the pre-test, revisions and modifications were made. During subsequent visits and at discharge, each mother received health education regarding the outcomes of interest.

### Data processing and analysis

Data entry was conducted using EpiData version 4.6.0.6, and subsequently, the data were exported to STATA version 17 for analysis. Descriptive statistics such as frequency tables, percentages, and median were utilized to describe the data. The log-rank test, Kaplan–Meier failure curve, and median time to death were computed. Various models, including the Cox proportional hazards model and three parametric models (Exponential, Weibull, and Gompertz), were applied to identify predictors of mortality among neonates admitted with sepsis. The Cox proportional hazards regression model emerged as the better model based on the lowest AIC score.

The proportional hazard assumption was evaluated through a log-log test (categories of survivorship difference). Statistical differences in survival were tested using the log-rank test (*p*-value <0.005). Additionally, time dependence and overall goodness of fit were assessed through Schoenfeld residual global tests (*χ*^2^ = 8.20, *p*-value = 0.098), indicating no violation of the assumption and suggesting the absence of time dependence. Consequently, the overall goodness of fit was met (Schoenfeld residual global tests >0.05). To identify predictors of time to death, we established both a bivariable and multivariable proportional hazard regression model. Variables with a *p*-value of less than 0.25 in the bivariable analysis were included in the multivariable analysis. In the multivariable analysis, we determined the adjusted hazard ratio (AHR) with a 95% confidence interval (CI), considering statistical significance at a *p*-value less than 0.05.

## Result

### Socio-demographic characteristics

In this study, 306 mother-neonate pairs participated. The majority of newborn mothers were aged between 26 and 30, comprising 110 (35.9%) of the total. Urban residents accounted for 161 (52.6%) of the sample, while 224 (73.2%) were housewives. Among the neonates, 171 (55.9%) were male. The mean age of the mothers was 28.3 years (SD ± 5.7), and the neonates' mean age was 3.7 days (SD ± 5.0) (Table 1).

**Table 1:** Sociodemographic characteristics of mothers and neonates admitted in NICU of comprehensive hospitals in northeastern, Ethiopia, 2023.

Variables	Frequency	Percent (%)
Maternal age		
18–20 years	18	5.9%
21–25	98	32%
26–30	110	35.9%
31–35	41	13.5%
36–40	29	9.6%
41–49	10	3.2%
Maternal Residence		
Urban	161	52.6%
Rural	145	47.4%
Educational status		
Unable read and write	79	25.8%
Primary school	73	23.9%
Secondary school	89	29.1%
College and above	65	21.2%
Occupation		
Housewife	224	73.2%
Civil servant	52	17%
Private work	30	9.8%
Neonate sex		
Male	171	55.9%
Female	135	44.1%
Neonate age		
1–3 days	225	73.7%
4–28 days	81	26.3%

### Obstetrics, labor, and delivery-related characteristics

The majority of mothers, 164 (53.6%), were multiparous; 300 (98%) received antenatal care (ANC) follow-up; 233 (76.1%) delivered at a hospital, and 198 (64.7%) had spontaneous vaginal delivery (SVD). Additionally, 91 (29.7%) experienced prolonged labor, 60 (19.6%) had premature rupture of membranes (PROM), 62 (20.3%) had a history of fever during labor, and 54 (17.6%) had pregnancy-induced hypertension (Table 2).

**Table 2:** Obstetrics, labor, and delivery related features of mothers whose neonates admitted in NICU of comprehensive hospitals of northeastern Ethiopia, 2023.

Variable	Frequency	Percent
Parity		
Primipara	142	46.4%
Multipara	164	53.6%
Had ANC follow up		
No	6	2%
Yes	300	98%
Frequency of ANC		
One times	21	7%
Two times	82	27.3%
Three times	109	36.3%
≥ 4 times	88	29.3%
History of fever during labor		
No	244	79.7%
Yes	62	20.3%
Onset of labor		
Spontaneous	288	94.1%
Induced	18	5.9%
Iron and folic acid supplement taken		
No	127	41.5%
Yes	179	58.5%
Prolonged labor		
No	215	70.3%
Yes	91	29.7%
Place of delivery		
Hospital	233	76.1%
Health center	56	18.3%
Home	17	5.6%
Mode of delivery		
SVD	198	64.7%
Instrumental delivery	21	6.9%
CS	87	28.4%
Had PROM		
No	246	80.4%
Yes	60	19.6%
History of diagnosed chorioamnionitis		
No	260	85.0%
Yes	46	15.0%
History of STI during pregnancy		
No	304	99.3%
Yes	2	0.7%
Had pregnancy induced hypertension		
No	252	82.4%
Yes	54	17.6%
Presence of maternal chronic illness		
No	297	97.1%
Yes	9	2.9%
Had placental abnormality		
No	303	99.0%
Yes	3	1.0%
HIV status		
Negative	218	71.2%
Positive	8	2.6%
Unknown status	80	26.1%
VDRL result		
Non-reactive	227	74.2%
Reactive	2	0.7%
Unknown status	77	25.2%
Hepatitis status		
Non-reactive	198	64.7%
Reactive	5	1.6%
Unknown status	103	33.7%

### Newborn medical problems and diagnostic features

The majority of neonates, 183 (59.8%), were term newborns; 77 (25.2%) experienced birth asphyxia, with only 48 (63.2%) receiving resuscitation. Additionally, upon admission, most newborns exhibited tachypnea (162 or 52.9%), respiratory distress syndrome (RDS) (179 or 58.5%), hypothermia (187 or 61.1%), and were unable to breastfeed (273 or 89.2%). Moreover, 263 (85.9%) of the Gram stain results were positive, and 228 (74.8%) were diagnosed with early-onset sepsis. Besides sepsis, neonates also experienced hypoglycemia (74 or 24.2%), meconium aspiration syndrome (MAS) (53 or 17.3%), perinatal asphyxia (PNA) (72 or 23.5%), and birth injuries (21 or 6.9%) (Table 3).

**Table 3:** Basic characteristics, medical problems and diagnostic features of neonates with sepsis admitted in NICU of comprehensive hospitals in northeastern Ethiopia, 2023.

Newborn clinical features	Status	
	Death	Censored
Gestational age classifications		
Term (37–42 weeks)	43 (41.3%)	140 (69.3%)
Preterm (<37 weeks)	58 (55.8%)	55 (27.2%)
Post term (>42 weeks)	3 (2.9%)	7 (3.5%)
Birth weight classifications		
Normal weight (≥2,500 g)	31 (29.8%)	133 (65.8%)
Low birth weight (<25,000 g)	73 (70.2%)	69 (34.2%)
APGAR score at 1st minute		
<7	35 (33.6%)	47 (23.4%)
≥7	69 (66.4%)	155 (76.6%)
APGAR score at fifth minute		
<7	31 (29.7%)	44 (21.8%)
≥7	73 (70.3%)	158 (78.2%)
Had asphyxia at birth		
No	72 (69.2%)	157 (77.7%)
Yes	32 (30.8%)	45 (22.3%)
Was neonate resuscitated		
No	16 (50%)	12 (27.3%)
Yes	16 (50%)	32 (72.7%)
Had convulsion		
No	82 (78.8%)	182 (90.1%)
Yes	22 (21.2%)	20 (9.9%)
Respiratory rate status		
Tachypnea (>60 breath/min)	62 (59.6%)	100 (49.5%)
Normal breathing rate (30–60 beath/min)	42 (40.4%)	99 (49.0%)
Bradypnea (<30 breath/min)	0 (0%)	3 (1.5%)
Oxygen saturation		
≥90%	32 (30.8%)	116 (57.4%)
<90%	72 (69.2%)	86 (42.6%)
Respiratory distress syndrome		
No	19 (18.3%)	108 (53.5%)
Yes	85 (81.7%)	94 (46.5%)
Heart rate status		
Normal rate	59 (56.7%)	137 (67.8%)
Tachycardia	44 (42.3%)	63 (31.2%)
Bradycardia	1 (1.0%)	2 (1.0%)
Umbilical infection		
No	103 (99%)	200 (99%)
Yes	1 (1%)	2 (1%)
Temperature status		
Normal (36.5–37.5°C)	28 (26.9%)	69 (34.2%)
Hyperthermia (>37.5°C)	8 (7.7%)	14 (6.9%)
Hypothermia (<36.5°C)	68 (65.4%)	119 (58.9%)
Being lethargic or unconscious		
No	51 (49%)	50 (24.8%)
Yes	53 (51%)	152 (75.2%)
Reduced movement		
No	27 (26%)	87 (43.1%)
Yes	77 (74%)	115 (56.9%)
Not able to breastfeed		
No	7 (6.7%)	26 (12.9%)
Yes	97 (93.3%)	176 (87.1%)
Diagnosed with hypoglycemia		
No	75 (72.1%)	157 (77.7%)
Yes	29 (27.9%)	45 (22.3%)
Diagnosed with MAS		
No	86 (82.7%)	167 (82.7%)
Yes	18 (17.3%)	35 (17.3%)
Diagnosed with jaundice		
No	78 (75%)	163 (80.7%)
Yes	26 (25%)	39 (19.3%)
Gram stain		
Gram positive	91 (87.5%)	172 (85.1%)
Gram negative	13 (12.5%)	30 (14.9%)
Types of sepsis diagnosed		
EONS	80 (77.7%)	148 (73.3%)
LONS	23 (22.3%)	54 (26.7%)
Neonates diagnosed with PNA		
No	79 (76.0%)	155 (76.7%)
Yes	25 (24.0%)	47 (23.3%)
Neonates born with congenital anomalies		
No	91 (87.5%)	185 (91.6%)
Yes	13 (12.5%)	17 (8.4%)
Neonates diagnosed with birth injury		
No	97 (93.3%)	188 (93.1%)
Yes	7 (6.7%)	14 (6.9%)

### Management and complication of neonates with sepsis

Of the neonates diagnosed with sepsis, 111 (36.3%) developed anemia, while only 3 (1%) experienced septic shock, 29 (9.5%) suffered from necrotizing enterocolitis (NEC), and 8 (2.6%) had severe thrombocytopenia. The majority, 230 (75.2%), received respiratory support, with 188 (81%) administered intranasal oxygen (INO2), 64 (20.9%) undergoing phototherapy, and 184 (60.1%) receiving ampicillin and gentamicin. Additionally, 276 (90.2%) received maintenance fluid, 50 (16.3%) received calcium gluconate, 109 (35.7%) received thermal care, and 51 (16.7%) underwent whole blood or platelet transfusion (Table 4).

**Table 4:** Management and complications neonates with sepsis admitted in NICU of comprehensive hospitals in northeastern Ethiopia, 2023.

Variables	Status	
	Death	Censored
Anemia		
No	70 (67.3%)	125 (61.9%)
Yes	34 (32.7%)	77 (38.1%)
Meningitis		
No	103 (99%)	198 (98)
Yes	1 (1%)	4 (2%)
Septic shock complications		
No	102 (98.1%)	201 (99.5%)
Yes	2 (1.9%)	1 (0.5%)
NEC development		
No	85 (81.7%)	192 (95%)
Yes	19 (18.3%)	10 (5%)
NG tube feeding		
No	41 (39.4%)	132 (65.3%)
Yes	63 (60.6%)	70 (34.7%)
Had respiratory support		
No	7 (6.7%)	69 (34.2%)
Yes	97 (93.3%)	133 (65.8%)
Type of respiratory support		
INO2	64 (65.3%)	124 (92.5%)
CPAP	34 (34.7%)	10 (7.5%)
Received phototherapy treatment		
No	80 (76.9%)	162 (80.2%)
Yes	24 (23.1%)	40 (19.8%)
Had maintenance fluid		
No	7 (6.7%)	23 (11.4%)
Yes	97 (93.3%)	179 (88.6%)
Had calcium gluconate		
No	78 (75%)	178 (88.1%)
Yes	26 (25%)	24 (11.9%)
Had antiseizure		
No	86 (82.7%)	189 (93.6%)
Yes	18 (17.3%)	13 (6.4%)
Has received radiant warmer care		
No	54 (52.4%)	142 (70.3%)
Yes	49 (47.6%)	60 (29.7%)
Transfusion had given		
No	79 (76%)	176 (87.1%)
Yes	25 (24%)	26 (12.9%)

### Time to death with neonatal sepsis

The cumulative incidence of mortality among neonates admitted with sepsis was 34% (95% CI: 28.9%–39.5%). The neonatal mortality rate was 51 (95% CI: 42.1, 62) per 1,000 neonates admitted to the intensive care units with sepsis, based on a total of 1,854 person-day observations. Moreover, the majority of deaths (80, 77.7%) occurred within the first three days of hospitalization, with a median time to death of 13 days (IQR = 5–23 days).

**Figure 1:**
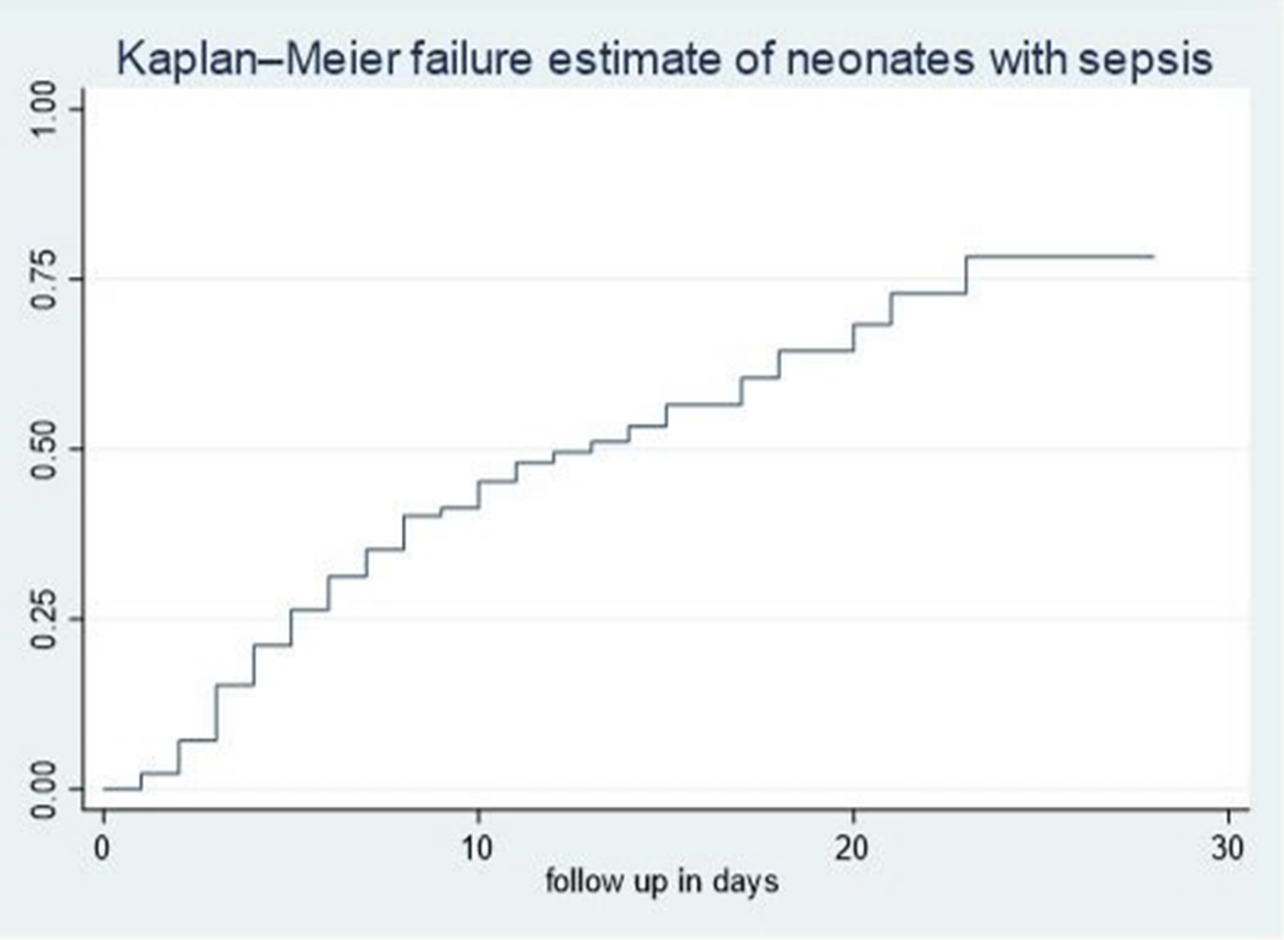
Overall Kaplan–Meier failure estimates of neonates with sepsis admitted in NICU of comprehensive hospitals in northeastern hospitals, 2023.

The cumulative failure rate among septic neonates was initially low on the first day after admission but increased steadily with follow-up time up to 28 days of age. Specifically, the cumulative failure rate of septic neonates at the end of the first, third, seventh, fourteenth, twentieth, and twenty-eighth days was 2.35%, 15.93%, 37.11%, 56.1%, 70.63%, and 79.86%, respectively. The hazard of death increases as the duration of hospitalization extends (Figure 1).

### Time to death comparisons for various categorical variables

**Figure 2:**
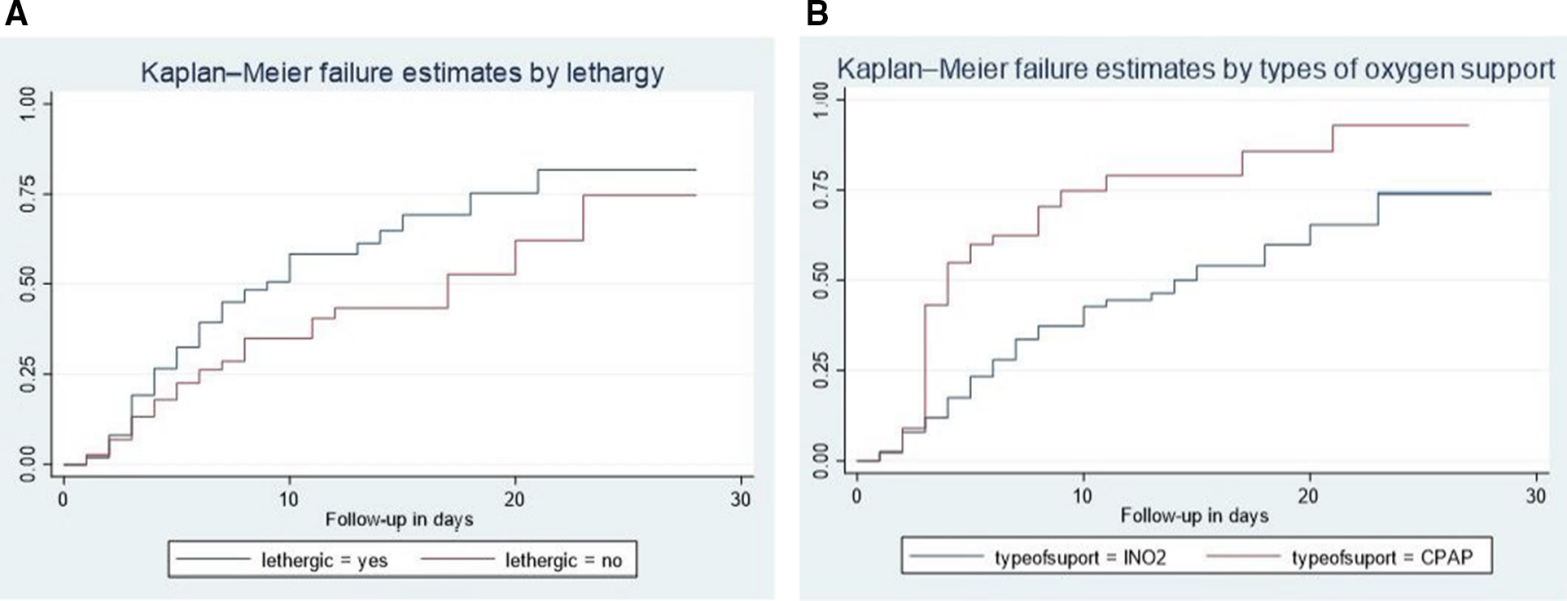
Kaplan–Meier failure curve showing different hazards across different categories of the covariates (lethargy (**A**), newborn counseling care (**B**), and types of oxygen support.

In our study, lethargic neonates exhibited a higher risk of mortality compared to non-lethargic neonates. The median hazard time to death for lethargic neonates was 9 days, significantly lower than the 17-day median observed for non-lethargic neonates (95% CI: 1.2–2.6). Additionally, the cumulative incidence rate of neonatal mortality stood at 7.2 per 100 among lethargic neonates with sepsis admitted to NICU comprehensive specialized hospitals. The difference in survival rates was statistically significant with a *p*-value of 0.003 during the log-rank test (Figure 2A).

CIn addition, neonates who received CPAP treatment experienced a shorter median time to death compared to those who received INO2. The median hazard time to death for neonates who underwent CPAP therapy was 4 days, significantly lower than the 14-day median observed for neonates treated with INO2 (95% CI: 1.3–3.4). Furthermore, the cumulative incidence rate of neonatal mortality among neonates treated with CPAP and admitted to NICU comprehensive specialized hospitals with sepsis was 10.1 per 100. The difference in survival rates was statistically significant with a *p*-value of 0.000 during the log-rank test (Figure 2B).

### Model fitness

**Figure 3:**
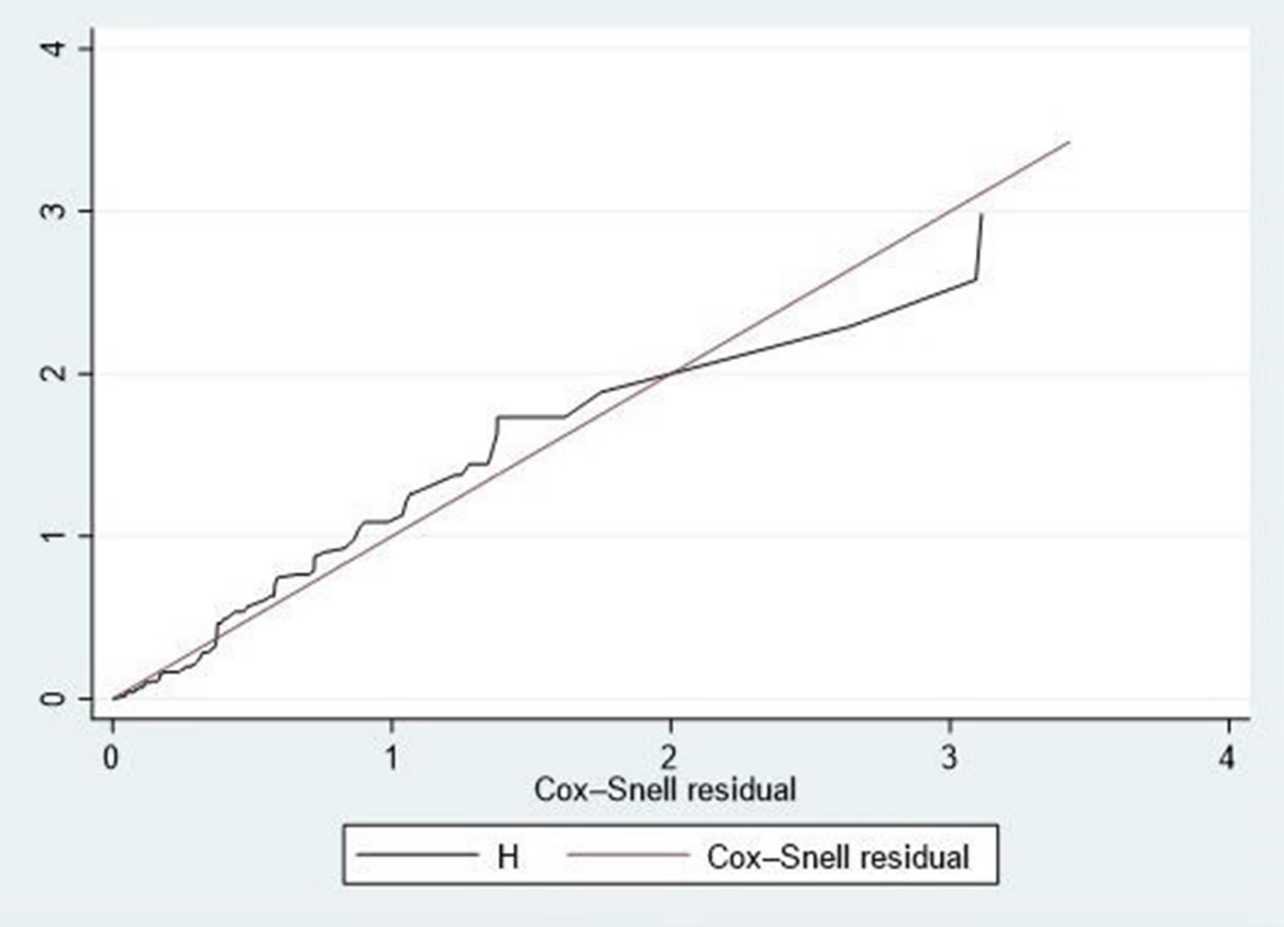
Cox-snell residual test for the overall model fitted for time to death among neonates admitted with neonatal sepsis.

The overall goodness of fit was assessed using Schoenfeld residual global tests (*χ*^2^ = 8.20, *p*-value = 0.098), indicating a good fit for the model (the Schoenfeld residual global test was not statistically significant, >0.05). Furthermore, Cox-Snell residual plots were employed to evaluate the overall goodness-of-fit of the Cox proportional hazard regression model. The line representing the Cox-Snell residual of the Cox proportional hazard regression model closely aligned with the 45° straight line. As depicted in Figure 3, this indicates a well-fitted model.

### Predictors of time to death among neonate with sepsis

Following the bivariable proportional hazard regression analysis, factors with *p*-values less than 0.25 were included in the multivariable proportional hazard regression. The analysis revealed that tachypnea, respiratory distress syndrome (RDS), lethargy, preterm birth, subgaleal hemorrhage, CPAP usage, home delivery, absence of newborn care counseling, and low platelet count were predictors of neonatal sepsis mortality (Table 5).

**Table 5:** Predictors of time to death among neonates with sepsis admitted in NICU of comprehensive hospitals in northeastern hospitals, 2023.

Factors	Status		CHR (95% CI)	AHR (95% CI)	*p*-value
	Death	Censored			
Having intrapartum fever history					
No	72	172	1	1	1
Yes	32	30	1.54 (1.01, 2.34)*	1.45 (.94, 2.25)	.091
Place of delivery					
Health center	13	43	1	1	1
Hospital	83	150	1.5 (.83, 2.7)	1.4 (.77, 2.54)	.272
Home	8	9	2.5 (1.03, 6.1)*	**2.63 (1.1, 6.4)** **	.033
Gestational age					
Term birth	43	140	1	1	1
Preterm birth	58	55	2.1 (1.4, 3.1)*	**1.8 (1.2, 2.7)** **	.005
Post term birth	3	7	1.6 (.5, 5.1)	1.9 (.6, 6.1)	.309
Respiratory status					
Bradypnea	0	3	1	1	1
Tachypnea	62	100	6.4 (4.32, 9.6)*	**6.2 (1.5, 9.7)** **	.000
Normal breathing rate	42	99	2.5 (1.6, 3.8)	1.3 (1.1, 3.7)	.456
Oxygen saturation					
≥90%	32	116	1	1	1
<90%	72	86	1.7 (1.1, 2.5)*	1.3 (.8, 2.1)	.298
Respiratory distress syndrome					
No	19	108	1	1	1
Yes	85	94	2.4 (1.5, 4.1)*	**2.1 (1.3, 3.5)** **	.005
Lethargic					
No	51	50	1	1	1
Yes	53	152	1.8 (1.2, 2.6)*	**1.8 (1.2, 2.6)** **	.000
Platelet count					
Increased	14	50	1	1	1
Normal	39	84	1.4 (.75, 2.54)	2.9 (1.3, 6.4)	.078
Had thrombocytopenia	51	68	1.9 (1.1, 3.4)*	**5.9 (2.3, 8.6)** **	.000
Types of birth injury					
Caput succedaneum	0	1	1	1	1
Cephalohematoma	3	3	2.8 (1.3, 6.1)	6.4 (1.1, 9.72)	.089
Subgalea hemorrhage	4	10	4.5 (2.3, 9.2)*	**1.8 (1.1, 3.9)** **	.003
Types of respiratory support					
INO2	64	124	1	1	1
CPAP	34	10	2.3 (1.5, 3.5)*	**2.1(1.3, 3.4)** **	.003

* Stands for statistical in crude hazard ratio.

** Stands for statistical significance in adjusted hazard ratio.

During the follow-up period, neonates diagnosed with sepsis who were born preterm experienced roughly a two-fold shorter time to death (CI: 1.2–2.7) compared to those born at full term. Similarly, newborns diagnosed with respiratory distress syndrome (RDS) upon admission had nearly twice the risk of a shorter time to death (CI: 1.3–3.5) compared to those without RDS. Additionally, newborns delivered at home faced an almost threefold shorter time to death (CI: 1.1–6.4) compared to those delivered in health centers. Likewise, neonates treated with CPAP had twice the risk of a shorter time to death (CI: 1.3–3.4) compared to those treated with INO2. Furthermore, newborns experiencing complications like a low platelet count had nearly six times the risk of a shorter median time to death (95% CI: 2.3–8.6) compared to those with normal platelet counts.

## Discussion

The current study aimed to evaluate the time to death of neonates with sepsis and its predictors among those admitted to the NICU of comprehensive hospitals in northeastern Ethiopia. The neonatal mortality rate was 51 (95% CI: 42.1, 62) per 1,000 neonates admitted to the intensive care units with sepsis, based on a total of 1,854 person-day observations. This figure was lower compared to rates in low-income nations (111.77), low-middle-income countries (70.06), Western Sub-Saharan Africa (63.84), and Northern Ethiopia (63 per 1,000 live births) (5). However, it was higher compared to rates in high-income nations (1.53) and middle-income countries (35.82) per 1,000 live births (5), as well as in Brazil (18.6) (21), southern Ethiopia (14.57) (7), and Northwestern Ethiopia (28) per 1,000 live births (8). The observed differences could stem from variations in sample sizes, as studies with larger sample sizes tend to exhibit lower mortality rates compared to those with smaller samples. Consequently, the mortality rate in our study might appear higher than in studies with smaller samples, yet lower than those with larger ones. Additionally, differences in study settings (urban, rural, or mixed) play a significant role. Previous studies either focused on neonates from major urban centers or remote regions, resulting in lower mortality rates in urban areas and higher rates in rural ones. In contrast, our study encompassed both settings, potentially yielding mortality rates higher than those observed in remote areas yet lower than those in urban-focused studies. Furthermore, studies conducted in primary and general hospitals typically reported higher infant mortality rates than those in tertiary and comprehensive hospitals. Consequently, our study's mortality rate may be higher than those conducted in higher-tier hospitals but lower than those in primary and general hospitals. Similarly, variations in socioeconomic status between nations contribute to differences in mortality rates.

The median time to death in the current study was 13 days (IQR = 5–23 days), a figure comparable to the findings of a study conducted in southern Ethiopia, which reported a median time of 13 days (IQR = 4–14 days) (7). However, studies conducted in northwest Ethiopia and Brazil revealed shorter median times to death, at 6 days (IQR = 3–8 days) (8) and 8 days (IQR = 3–21 days) (21), respectively. This discrepancy could be attributed to differences in the quality of care, clinical setup, competence of healthcare personnel, and the availability of medications. These findings suggest that a higher median mortality rate might be preventable with the implementation of specific measures to enhance the quality of care, provide training for healthcare providers, and ensure the availability of essential drugs.

Preterm neonates diagnosed with sepsis exhibit a two-fold shorter time to death compared to term babies. This trend is consistent with findings from a multisite prospective study conducted across low- and middle-income nations (22), as well as global research conducted in China, Brazil, India, and northwest Ethiopia (8, 21, 23, 24, 25). A plausible explanation is that premature newborns are more susceptible to infections due to the immaturity of vital organs and their immune systems (18). Furthermore, premature infants often require specialized continuous care, including surfactant drug administration, cooling therapy, and advanced support such as extracorporeal membrane oxygenation (4). However, critical services like these are often limited in developing countries (24, 25), leading to an increased risk of hazards and resulting in a shorter time to death among neonates affected by sepsis.

In the current study, neonates diagnosed with respiratory distress syndrome (RDS) were found to have an increased risk of hazards and a shorter time to death from sepsis compared to their counterparts. This observation aligns with findings from previous studies conducted globally, in China, Northwest Ethiopia, and Felegehiwot Comprehensive Hospital (8, 24, 25, 26). The prevalence of RDS as a complication among preterm neonates is attributed to respiratory organ immaturity (18). Consequently, neonates with RDS are more susceptible to developing infections such as sepsis, thereby worsening the prognosis of sepsis. Additionally, the scarcity of RDS support care, including surfactant medications and advanced therapies like extracorporeal membrane oxygenation, in low-income countries (4), particularly in Ethiopia, exacerbates the situation. As a result, the presence of RDS worsens the prognosis of sepsis, leading to a shorter median time for neonatal mortality due to sepsis.

Neonates with sepsis who received CPAP treatment showed a higher risk of death and a shorter time to mortality compared to those treated with INO2. This trend is consistent with findings from studies conducted globally and in China (24, 25). The choice of respiratory support machinery is primarily determined by the gestational age of the neonates. In Ethiopia, for instance, CPAP is administered to very preterm infants (less than 32 weeks), mid preterm babies (32–34 weeks), and occasionally to late preterm neonates (34–36 weeks) experiencing complications related to respiratory distress syndrome (RDS). On the other hand, INO2 is typically reserved for term infants or late preterm babies requiring respiratory assistance. It's important to note that neonates receiving CPAP or INO2 may present with a spectrum of illnesses ranging from mild to severe, all necessitating respiratory support. Thus, the decision to utilize different types of respiratory support is predominantly influenced by the gestational age of the neonate rather than the severity of their illness. Another plausible explanation within our study context is the utilization of locally made CPAP accessories, often crafted from cut water container plastics. During routine care procedures, these materials might become contaminated, inadvertently exposing neonates to sepsis.

Home-delivered babies exhibited a nearly threefold shorter time to death compared to facility deliveries, a trend consistent with studies conducted in India (23, 27). The plausible explanation lies in the risks associated with home delivery, where newborns may be exposed to sepsis due to several factors: assistance by non-medical personnel, inadequate sterility of delivery instruments and surroundings, and an increased likelihood of cord care malpractice (18). Consequently, neonates face a shorter time to death from sepsis. This underscores the importance of strengthening the culture of institutional delivery.

Neonates with a decreased platelet count during hospitalization died sooner than their counterparts, a trend consistent with findings from Turkey (28). This could be attributed to the fact that a neonate with sepsis and a decreased platelet count often experiences significant internal bleeding complications, leading to conditions such as renal failure and multi-organ failure (4, 18, 24, 25). Consequently, this exacerbates the prognosis of sepsis and contributes to a shorter time to death.

Neonates with sepsis exhibiting lethargy and tachypnea experienced earlier mortality, aligning with studies conducted in northwest Ethiopia (8, 15). Lethargy and tachypnea during admission often lead to respiratory failure and hypoglycemia issues. These complications exacerbate the prognosis of sepsis (18, 24, 25), resulting in a shorter lifespan for affected neonates.

### Limitations and strengths of the study

The authors utilized a standardized tool for data collection and adopted a multi-center approach with an adequate sample size. Despite the gold standard for diagnosing sepsis being culture, resource limitations in diagnostics and therapeutics prevented its use for diagnosis in this study. Consequently, clinical diagnosis became pivotal, potentially leading to variations in physician diagnoses of clinical sepsis. This variance might influence the incidence of neonatal sepsis mortality. Furthermore, researchers may consider employing a cause-specific mortality estimate (competing risk survival model) to pinpoint specific causes of death in future studies.

## Conclusion

In this study, the incidence of neonatal mortality due to sepsis was alarmingly high, necessitating immediate intervention. To mitigate neonatal sepsis-related deaths, enhancing the quality of care in NICU settings is crucial. Additionally, it's imperative to improve infection prevention measures during CPAP procedures or consider avoiding locally made CPAP accessories. Furthermore, behavioral change and health promotion activities should highlight the risks associated with home delivery, such as neonatal sepsis mortality. Efforts aimed at reinforcing the culture of institutional delivery are also essential.

## Data Availability

The raw data supporting the conclusions of this article will be made available by the authors, without undue reservation.

## References

[1] Atif M Zia R Malik I Ahmad N Sarwar S. Treatment outcomes, antibiotic use andits resistance pattern among neonatal sepsis patients attending Bahawal victoria hospital, Pakistan. PLoS One. (2021) 16(1):e0244866. 10.1371/journal.pone.0244866 33439876 PMC7806133

[2] Hematyar M Najibpour R Bayesh S Hojjat A Farshad A. Assessing the role of clinical manifestations and laboratory findings in neonatal sepsis. Arch Pediatr Infect Dis. (2017) 5(1):e29985. 10.5812/pedinfect.29985

[3] Shane AL Sánchez PJ Stoll BJ. Neonatal sepsis. Lancet. (2017) 390(10104):1770–80. 10.1016/S0140-6736(17)31002-4 28434651

[4] Li J Xiang L Chen X Li S Sun Q Cheng X Global, regional, and national burden of neonatal sepsis and other neonatal infections, 1990–2019: findings from the global burden of disease study 2019. Eur J Pediatr. (2023) 182:2335–43. 10.1007/s00431-023-04911-7 36879151

[5] Li J Shen L Qian K. Global, regional, and national incidence and mortality of neonatal sepsis and other neonatal infections, 1990–2019. Front Public Health. (2023) 11:1139832. 10.3389/fpubh.2023.1139832 36998277 PMC10043440

[6] Ethiopian Public Health Institute (EPHI) [Ethiopia] and ICF. Ethiopia Mini Demographic and Health Survey 2019: Final Report. Rockville, Maryland, USA: EPHI and ICF (2021).

[7] Dessu S Habte A Melis T Gebremedhin M. Survival status and predictors of mortality among newborns admitted with neonatal sepsis at public hospitals in Ethiopia. Int J Pediatr. (2020) 2(3):1–9. 10.1155/2020/8327028 PMC7527886 33029155

[8] Abiy SA Animut Y Ambaw WM Aragaw GM Rade BK. Incidence of death and its predictors among neonates admitted with sepsis in referral hospitals, northwest Ethiopia, a prospective cohort study. Front Pediatr. (2023) 11:1129924. 10.3389/fped.2023.1129924 37124184 PMC10133692

[9] UNICEF, WHO, World Bank, United Nations. Levels and trends in child mortality report 2018. Estimates developed by the United Nations Inter-Agency Group for Child Mortality Estimation. New York City (NY): UNICEF. (2019).

[10] Ranjeva SL Warf BC Schiff SJ. Economic burden of neonatal sepsis in sub-Saharan Africa. BMJ Glob Health. (2018) 3:e000347. 10.1136/bmjgh-2017-000347 29564153 PMC5859806

[11] Bayih WA Ayalew MY Chanie ES Abate BB Alemayehu SA Belay DM The burden of neonatal sepsis and its association with antenatal urinary tract infection and intra-partum fever among admitted neonates in Ethiopia: a systematic review and meta-analysis. Heliyon. (2021) 7(2):1–14. 10.1016/j.heliyon.2021.e06121 PMC7887389 33644445

[12] Berardi A Sforza F Baroni L Spada C Ambretti S Biasucci G Epidemiology and complications of late-onset sepsis: an Italian area-based study. PLoS One. (2019) 14(11):e0225407. 0225410.0221371/journal.pone.0225407 31756213 10.1371/journal.pone.0225407 PMC6874360

[13] Gebremedhin D Berhe H Gebrekirstos K. Risk factors for neonatal sepsis in public hospitals of Mekelle city, North Ethiopia, 2015: unmatched case control study. PLoS One. (2016) 11(5):e0154798. 10.1371/journal.pone.0154798 27163290 PMC4862626

[14] Murthy S Godinho MA Guddattu V Lewis LES Nair NS. Risk factors of neonatal sepsis in India: a systematic review and meta-analysis. PLoS One. (2019) 14(4):e0215683. 12.1371/journal.pone.0215683 31022223 10.1371/journal.pone.0215683 PMC6483350

[15] Oumer M Abebaw D Tazebew A. Time to recovery of neonatal sepsis and determinant factors among neonates admitted in public hospitals of central Gondar zone, Northwest Ethiopia, 2021. PLoS One. (2022) 17(7):e0271997. 10.1371/journal.pone.0271997 35900981 PMC9374017

[16] Wale A Chelkeba L. Treatment outcome and associated factors of neonatal sepsis at Mizan-Tepi university teaching hospital, south west Ethiopia: a prospective observational study. Pediatric Health Med Ther. (2021) 12:467–79. 10.2147/PHMT.S322069 34539194 PMC8443800

[17] Federal Democratic Republic of Ethiopia Ministry of Health. National strategy for newborn and child survival in Ethiopia, 2015/16–2019/20. Maternal and Child Health Directorate Federal Ministry of Health. Addis Ababa. (2015).

[18] Ethiopia Federal Ministry of Health. Neonatal intensive care unit (NICU) management protocol. Federal Ministry of Health. (2021).

[19] Ethiopian Public Health Institute. National technical guidance for maternal and prenatal death surveillance and response (2017).

[20] Mengesha HG Sahle BW. Cause of neonatal deaths in Northern Ethiopia: a prospective cohort study. BMC Public Health. (2017) 17(1):1–8. 10.1186/s12889-016-3979-8 28077109 PMC5225539

[21] Freitas FT Araujo AF Melo MI Romero GA. Late-onset sepsis and mortality among neonates in a Brazilian intensive care unit: a cohort study and survival analysis. Epidemiol Infect. (2019) 147:1–7. 10.1017/S095026881900092X PMC6624867 31364533

[22] Milton R Gillespie D Dyer C Taiyari K Carvalho MJ Thomson K Neonatal sepsis and mortality in low-income and middle-income countries from a facility-based birth cohort: an international multisite prospective observational study. Lancet Glob Health. (2022) 10(5):e661–72. 10.1016/S2214-109X(22)00043-2 35427523 PMC9023753

[23] Murthy S Godinho MA Guddattu V Lewis LE Nair NS. Risk factors of neonatal sepsis in India: a systematic review and meta-analysis. PLoS One. (2019) 14(4):e0215683. 10.1371/journal.pone.0215683 31022223 PMC6483350

[24] Russell NJ Stöhr W Plakkal N Cook A Berkley JA Adhisivam B Patterns of antibiotic use, pathogens, and prediction of mortality in hospitalized neonates and young infants with sepsis: a global neonatal sepsis observational cohort study (NeoOBS). PLoS Med. (2023) 20(6):e1004179. 10.1371/journal.pmed.1004179 37289666 PMC10249878

[25] Wang L Li JH Yu YH Huang L Huang XY Fan XF Initial respiratory support modality and outcome in preterm infants with less than 32 weeks of gestation in China: a multicentre retrospective cohort study. Paediatr Perinat Epidemiol. (2022) 36:390–8. 10.1111/ppe.12801 34431114 PMC9291106

[26] Tewabe T Mohammed S Tilahun Y Melaku B Fenta M Dagnaw T Clinical outcome and risk factors of neonatal sepsis among neonates in felege hiwot referral hospital, Bahir Dar, Amhara regional state, North West Ethiopia 2016: a retrospective chart review. BMC Res Notes. (2017) 10:1–7. 10.1186/s13104-017-2573-1 28693597 PMC5504561

[27] Bulto GA Fekene DB Woldeyes BS Debelo BT. Determinants of neonatal sepsis among neonates admitted to public hospitals in central Ethiopia: unmatched case-control study. Glob Pediatr Health. (2021) 8:2333794X211026186. 10.1177/2333794X211026186 34212071 PMC8216335

[28] Turhan EE Gürsoy T Ovalı F. Factors which affect mortality in neonatal sepsis. Turk Pediatri Ars. (2015) 50:170–5. 10.5152/TurkPediatriArs.2015.2627 26568693 PMC4629925

