# Supplementary material for: Time to death and its predictors among neonates admitted with sepsis in neonatal intensive care unit at comprehensive specialized hospitals in Northeast Ethiopia
Source: Front Pediatr. 2024 Apr 17;12:1366363. doi: 10.3389/fped.2024.1366363 (PMC11072712; doi:10.3389/fped.2024.1366363)
Supplement: Supplementary file 1 [file Datasheet1.pdf]

PART I. Sociodemographic factors			
No.	Question	Response	Skip
101	Age of mothers	-----------------------	
102	Place of residence	1. Urban 2. Rural	
103	Educational status	1.unable to read and write<br>2. primary school<br>3. secondary school<br>4.Tertiary education	
104	Occupation status	1.Housewife<br>2. Private work<br>3. Civil servant<br>4. others	
**PART II. Maternal health-related factors**			
105	Gravidity		
106	Parity		
107	Did the mother have an ANC follow-up for indexed neonate?	1. Yes 2. No	If 'no' skip to 109
108	If yes, what number of ANC visits?	______ times	
109	Did the mother have history of fever during your pregnancy of this neonate?	1. Yes<br>2. No	
110	Onset of labour	1. spontaneous 2. induced	
111	How long was the duration of labor?	----------- hours	
112	Where did you deliver your child?	1. Home<br>2. Heath center<br>3. Hospital	
113	What was the mode of delivery?	1. SVD<br>2. Assisted delivery<br>3. C/S	
114	PROM	1. Yes<br>2. No	
115	Duration of rupture of membrane	1. ---------hours<br>2. Not ruptured	
116	Did the amniotic fluid was foul smelling	1. Yes<br>2. No	
117	Did you have a history of UTI/STI during your pregnancy with this neonate? (VDRL test)	1. Yes<br>2. No<br>3. unknown	
118	HIV test	1. Yes<br>2. No<br>3. unknown	
119	Hepatitis test	1. Yes<br>2. No<br>3. unknown	
120	Pregnancy-induced HTN during pregnancy	1. Yes<br>2.No	

121	Presence of maternal chronic illness	1. Yes<br>2.No	
122	Placental abnormality	1. Yes<br>2.No	

**PART III: neonatal related factors**			
123	Sex of the neonate	1. Male 2. Female	
124	Age of neonate	------------ days	
125	Gestational age at birth	--------- weeks	
126	Weight of the neonate at birth in gram	--------- gm	
127	End of follow up weight	--------- gm	
128	Does the neonate was asphyxiated?	1. Yes 2. No	
129	Did the neonate was resuscitated?	1. Yes 2. No	
130	APGAR score at 1st minute and 5th minutes	------/10, ------/10	
**PART IV: Clinical criteria for the diagnosis of neonatal sepsis from observation/ chart review**			
		**Admission profile**	**End of follow up profile**
131	Convulsions/seizure	Yes ______ No ____	Yes ______ No ____
132	Respiratory rate > 60 breaths/min	Yes ______ No ____	Yes ______ No ____
133	Oxygen saturation	1.< 90% 2. ≥ 90%	1.< 90% 2. ≥ 90%
134	Respiratory distress syndrome	Yes ______ No ____	Yes ______ No ____
135	Heart rate	______	______
136	Redness around umbilicus extending to the skin	Yes ______ No ____	Yes ______ No ____
137	Temperature >37.7°C or <35.5°C	______	______
138	Lethargic or unconscious	Yes ______ No ____	Yes ______ No ____
139	Reduced movements	Yes ______ No ____	Yes ______ No ____
140	Not able to breast feed	Yes ______ No ____	Yes ______ No ____
141	Hypoglycemia	Yes ______ No ____	Yes ______ No ____
142	Jaundice	Yes ______ No ____	Yes ______ No ____
143	MAS	Yes ______ No ____	Yes ______ No ____

144	Bulging fontanels	Yes ______ No ____	Yes ______ No ____
**PART V:** Laboratory findings for the diagnosis of neonatal sepsis			
N.o	Type of laboratory finding		range
145	CBC profile WBC count	______	1. < 5000<br>2. 5000-20000<br>3. > 20,000
146	Platelet count(cell/mm3)	______	1. $<150 \times 10^3$<br>2. $150–450 \times 10^3$<br>3. 450×10^3
147	Hemoglobin/Hgb mg/dl	______	
148	Random blood sugar		
149	Gram stain	1. Yes 2. No	1. Positive<br>2. Negative
150	CSF analysis	1. Yes 2. No	1.Positive indicator<br>2. No indicator
151	Type of sepsis diagnosed	1. EONS 2. LONS	
152	Associated infection/ birth injury or other diagnosis	a) Meningitis 1. Yes 2. No<br>b) PNA 1. Yes 2. No<br>c) Septic shock 1. Yes 2. No<br>d) Congenital anomalies 1. Yes 2. No<br>e) NEC 1. Yes 2. No<br>f) Other ---- (specify)	
**PART VI:** Treatment pattern and its outcome			
153	NG tube feeding	1.Yes 2. No	
154	Respiratory help	1.Yes 2. No	
155	Is 'yes', the type of respiratory help	1. INO_2<br>2. CPAP<br>3. Other -------(specify)	
156	Is the child on phototherapy	1. Yes 2. No	
157	Iv-line medication	a) Ampicillin + gentamycin<br>b) Ampicillin + ceftriaxone<br>c) Cefatoxime + gentamycin<br>d) Vancomycin	
158	Co-administered drug along with antibiotics	a) PCM suppository<br>b) Calcium gluconate<br>c)Maintenance fluid 10%<br>d) Phenytoin or phenobarbitone<br>f) other………………….	

159	Duration of hospital stay	………………. days
160	Outcome after admission	1. Improved<br>2. Develop complication/ disability<br>3. Death<br>4. Referral/ transfer in<br>5. Refuse against medical treatment
