# Supplementary material for: Time to death and its predictors among neonates admitted with sepsis in neonatal intensive care unit at comprehensive specialized hospitals in Northeast Ethiopia
Source: Front Pediatr. 2024 Apr 17;12:1366363. doi: 10.3389/fped.2024.1366363 (PMC11072712; doi:10.3389/fped.2024.1366363)
Supplement: Supplementary file 2 [file Datasheet2.pdf]

Annex: Test of interaction by multicollinearity

Variables	VIF	1/VIF
Having intrapartum fever history	3.1	0.3225806452
Place of delivery	1.5	0.66666667
Gestational age	1.3	0.7692307692
Respiratory status	1.23	0.8130081301
Oxygen saturation	1.34	0.7462686567
Respiratory distress syndrome	1.56	0.641025641
Lethargic	1.4	0.7142857143
Platelet count	1.7	0.58882352941
Types of birth injury	1.78	0.5617977528
Types of respiratory support	1.83	0.5464480874
Transfusion had given	2.1	0.4761904762
Had antiseizure	3.2	0.3125
Has received radiant warmer care	2.87	0.348320557
Had respiratory support	2.1	0.4761904762
Neonates diagnosed with PNA	2.3	0.4347826087
Diagnosed with MAS	2.56	0.390625
Not able to breastfeed	2	0.5
Reduced movement	1.98	0.5050505051
Heart rate status	1.87	0.5347593583
Oxygen saturation	1.9	0.5263157895
Had convulsion	1.76	0.5681818182
Gestational age classifications	1.23	0.8130081301
Birth weight classifications	1.98	0.5050505051
Had PROM	2.1	0.4761904762
Onset of labor	2.9	0.3448275862
